# Rapid implementation of virtual clinics due to COVID-19: report and early evaluation of a quality improvement initiative

**DOI:** 10.1136/bmjoq-2020-000985

**Published:** 2020-05-21

**Authors:** Anthony William Gilbert, Joe C T Billany, Ruth Adam, Luke Martin, Rebecca Tobin, Shiv Bagdai, Noreen Galvin, Ian Farr, Adam Allain, Lucy Davies, John Bateson

**Affiliations:** 1 Therapies Department, Royal National Orthopaedic Hospital NHS Trust, Stanmore, UK; 2 School of Health Sciences, University of Southampton, Southampton, UK; 3 Operational Management, Royal National Orthopaedic Hospital NHS Trust, Stanmore, UK; 4 Improvement Team, Royal National Orthopaedic Hospital NHS Trust, Stanmore, UK

**Keywords:** quality improvement, telemedicine, PDSA

## Abstract

**Background:**

The COVID-19 outbreak has placed the National Health Service under significant strain. Social distancing measures were introduced in the UK in March 2020 and virtual consultations (via telephone or video call) were identified as a potential alternative to face-to-face consultations at this time.

**Local problem:**

The Royal National Orthopaedic Hospital (RNOH) sees on average 11 200 face-to-face consultations a month. On average 7% of these are delivered virtually via telephone. In response to the COVID-19 crisis, the RNOH set a target of reducing face-to-face consultations to 20% of all outpatient attendances. This report outlines a quality improvement initiative to rapidly implement virtual consultations at the RNOH.

**Methods:**

The COVID-19 Action Team, a multidisciplinary group of healthcare professionals, was assembled to support the implementation of virtual clinics. The Institute for Healthcare Improvement approach to quality improvement was followed using the Plan-Do-Study-Act (PDSA) cycle. A process of enablement, process redesign, delivery support and evaluation were carried out, underpinned by Improvement principles.

**Results:**

Following the target of 80% virtual consultations being set, 87% of consultations were delivered virtually during the first 6 weeks. Satisfaction scores were high for virtual consultations (90/100 for patients and 78/100 for clinicians); however, outside of the COVID-19 pandemic, video consultations would be preferred less than 50% of the time. Information to support the future redesign of outpatient services was collected.

**Conclusions:**

This report demonstrates that virtual consultations can be rapidly implemented in response to COVID-19 and that they are largely acceptable. Further initiatives are required to support clinically appropriate and acceptable virtual consultations beyond COVID-19.

**Registration:**

This project was submitted to the RNOH’s Project Evaluation Panel and was classified as a service evaluation on 12 March 2020 (ref: SE20.09).

## Introduction

The 2019 novel coronavirus (COVID 19) outbreak was first reported in Wuhan, China and reached the UK on 31 January 2020. On 11 March 2020, the WHO declared the COVID-19 virus a pandemic.^1^ COVID-19 mainly affects the upper respiratory tract, and associated clinical symptoms can be mild, severe or critical.^2^ The COVID-19 virus spreads primarily through droplets of saliva or discharge from the nose when an infected person coughs or sneezes. Social distancing measures have been established, with the UK public being placed on ‘lockdown’ from 23 March 2020^3^ to avoid transmission of the disease.

Physical attendance at outpatient clinics puts patients at risk of spreading COVID-19. Virtual consultations (VCs) are an important way for patients to access their care without this risk. There has been a surge in the interest for VC in response to COVID-19,^4 5^ with the National Health Service (NHS) in the UK releasing new information governance guidance for their use.^6^

The Royal National Orthopaedic Hospital (RNOH) is a specialist tertiary centre in Middlesex, UK. On average, 11 200 monthly face-to-face (F2F) consultations are held across two outpatient sites at the RNOH across a range of pathways.

The RNOH was actively developing virtual clinics prior to COVID-19 in line with the NHS Long Term Plan^7^ regarding reduction in F2F outpatient appointments. One of the project team had previously investigated the acceptability of VC^8 9^ and is actively researching this area.^10^ In November 2019, the operational management team agreed to use the VC platform Attend Anywhere with the licence for use granted on 27 February 2020. On 5 March 2020, in response to the growing COVID-19 crisis, a target of reducing F2F clinics to 20% of all outpatient attendances was set. The COVID-19 Action Team was established to support the delivery of this target.

In June 2018, the RNOH committed to applying the Institute for Healthcare Improvement (IHI) approach to quality improvement (QI) to all applicable change processes and established an improvement team to support delivery of this strategy. The IHI method is a formal approach which includes a clear process for thinking through, conducting and analysing the change ideas in a Plan-Do-Study-Act (PDSA) cycle.^11^ Normally the improvement team trains and coaches front-line teams to lead and deliver changes, but COVID-19 needed a rapid response, so experienced and skilled members of the improvement team joined the COVID-19 Action Team to support an improvement approach at pace.

The aim of the project was for 80% of all RNOH outpatient appointments to be delivered as VC (using Attend Anywhere or telephone) within 11 days of the target being set (target 80% from 16 March 2020). The secondary aim was to collect data to support the design of a substantive legacy of VC post-COVID-19.

## Methods

### Early enablement

The goal of 80% VC was set and communicated across all clinical staff. Clinical staff were asked to screen clinical lists and identify patients suitable for a VC (either a telephone consultation (TEL) or a video consultation (VID)) rather than F2F for the next 3 weeks. Software upgrades and hardware deployment began immediately and were completed within 24 hours across both sites at the RNOH.

### Process redesign and delivery support

The COVID-19 Action Team was established to rapidly implement VC across the RNOH. The multidisciplinary team consisted of operational management and strategists, a project manager, QI personnel, a clinical research fellow and data management support. The team provided a variety of skills and resources to facilitate implementation. Daily meetings were scheduled to identify processes that needed to be redesigned to facilitate VC. Issues and actions logs were created to identify and overcome obstacles to implementation.

### Measures

The overall approach to assess the impact of the intervention was straightforward: the percentage of patients undertaking VC (TEL or VID) compared with F2F. A combination of manual data collection of clinic lists and data taken from the RNOH patient management system was used to identify the proportion of patients undergoing VC. A simple, bespoke patient and clinician satisfaction questionnaire was developed to capture patient and clinician experience. This included the summative question ‘how satisfied were you with the virtual clinic?’ scored out of 100. These data collection methods were supplemented with informal observation and PDSA cycles.

### Analysis

Descriptive statistics were used to analyse quantitative data. A thematic analysis of qualitative data was used to illustrate the underlying reasons behind the quantitative data.

### Project registration

This project was submitted to the RNOH’s Project Evaluation Panel and was classified as a service evaluation on 12 March 2020 (ref: SE20.09).

## Results

Between 5 and 27 March, a large number of PDSA cycles were undertaken simultaneously across the five main areas shown in table 1 to support rapid implementation of both telephone and video virtual clinics. Coordination of activity and management of interdependencies were managed via the daily implementation group teleconference. PDSAs were considered, and appropriate action to expand, redesign or retest was agreed.

**Table 1:** PDSA cycle outcome

	PDSA group 1: administrative processes	PDSA group 2: clinician training and skills development	PDSA group 3: install technical infrastructure to deliver virtual clinics at scale	PDSA group 4: design and implementation of clinical pathways	PDSA group 5: patient and clinician experience
Number of cycles	12	9	8	3	4
Plan	To ensure standardised administrative processes are in place for effective booking and running of virtual clinics.	To understand clinical experience of virtual clinics across RNOH. To design ‘virtual clinic’ training tools available to all clinicians.	To equip all outpatient areas with the equipment required to run virtual clinics effectively at scale.	To ensure patients are able to access the required medication and diagnostics when attending clinics virtually.	To offer video and telephone appointments as an alternative consultation option.
Do	Map and redesign administrative booking process. Design new COF process to support virtual clinics from remote locations. Design and publication of standardised booking and patient communication tools. Admin leads allocated for each clinical pathway for refinement, approval and cascade of new processes. Manage the closure of Bolsover Street outpatient facility at RNOH.	Meet with teams experienced in telephone clinics and model processes. Clinical input into Attend Anywhere support tools. Trial with a clinician prior to going live and update support package. Allocation of daily ‘Floorwalkers’ to manage queries and opportunistically train within clinics. Daily clinic review feedback forms to inform troubleshooting tools and to refine coordination of outpatient clinics.	Licence approval for Attend Anywhere. Acquisition of headsets and webcams. Increase the number of external telephone lines from 60 to 200. Update all outpatient computers with the latest version of Chrome. Information leaflet regarding installing headsets and webcams. Infection control policy for sharing headsets. Create equipment log. Include technical support in floor-walker role.	Work with the pharmacy team to map the new medication pathway and SOP prior to ‘go live’. Design and implement new transport booking and cancellation process. Identify demand for essential diagnostics with clinical teams and design process to access as close to home as possible.	Call each patient to explain and offer alternatives. Design video appointment access details (specific to specialty) and patient guides. Created page on RNOH website with links to specialty waiting area as alternative access route. Establish process for monitoring patients waiting for video calls via admin screen.
Study	PDSAs coordinated by outpatient managers. Daily feedback enabled continuous improvement, with updates published to intranet folder and cascaded to front line. Recording of clinic type on appointment record to assist data collection.	Floor-walker roles important for troubleshooting. Face-to-face training more effective than training tools alone. Clinician blogs/stories shared via internal mail and social media. Training tools published to central intranet folder.	Floor-walker roles important to support staff members to set up. Process improved by gaining clinic list details 24 hours in advance. Excellent support from responsive information technology team enabled rapid acquisition of kit and updates required.	Pharmacy process in place with support from information governance lead. Transport booking process trialled on paper process, now electronic. Priority outpatient pathways being agreed and criteria for face-to-face/video/telephone clinics being reviewed with clinical leads to understand what demand will be for diagnostics moving forward.	Feedback collected via online survey at end of video appointments and paper ‘end of clinic reviews’, including patient feedback following telephone clinics. Data analysed daily to ensure real-time feedback so that any issues and suggestions are actioned quickly.
Act	*Implementation of new tools.* Clinic booking process SOP. COF process for remote working. Patient information leaflets. Development of an RNOH patient^19^ and generic NHS^20^ video. Patient telephone script and email confirmation templates. Support and executive ‘thank you’ to admin staff for achievement of this challenging role.	*Training tools in place.* Attend Anywhere patient video. Advice for conducting telephone consultations (based on ‘Human Factors’ principles). Attend Anywhere procedures and troubleshooting tools. Staff webinar.	All equipment and upgrades in place.	*New pathways in place.* Supply of medicines from RNOH virtual outpatient clinics SOP. Currently access to blood and diagnostics requiring face-to-face appointment. New pathways currently in negotiation with commissioning leads and NHS England.	Clinician and patient feedback mechanism in place. Ongoing data collection and more detailed analysis required to inform future practice and sustainability post-COVID-19.

COF, Clinic Outcome Form; NHS, National Health Service; PDSA, Plan-Do-Study-Act; RNOH, Royal National Orthopaedic Hospital; SOP, standard operating procedure.

**Figure 1:**
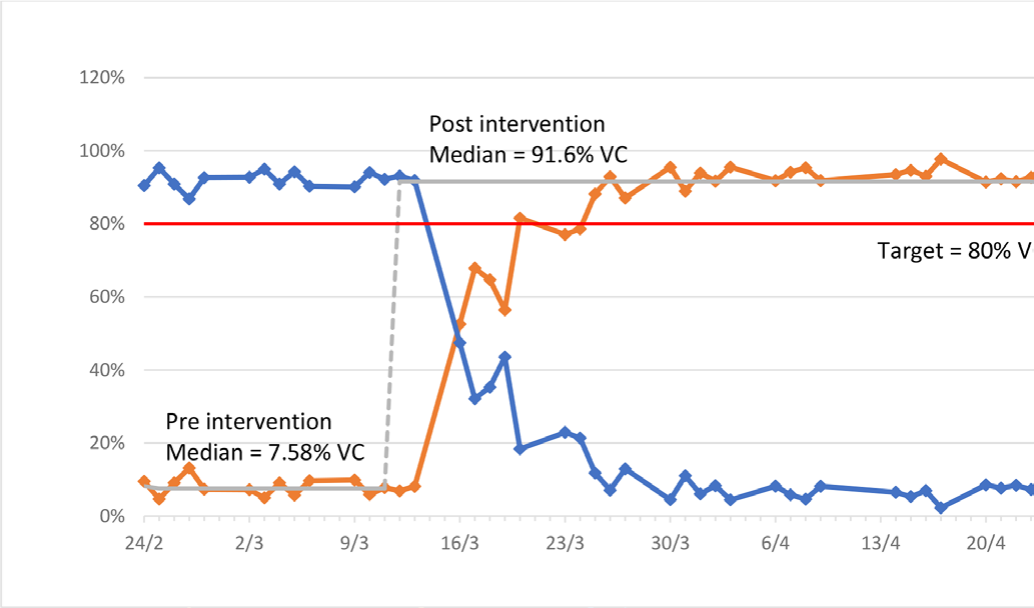
Run chart of virtual consultation (VC) and face-to-face (F2F) outpatient clinics.

**Figure 2:**
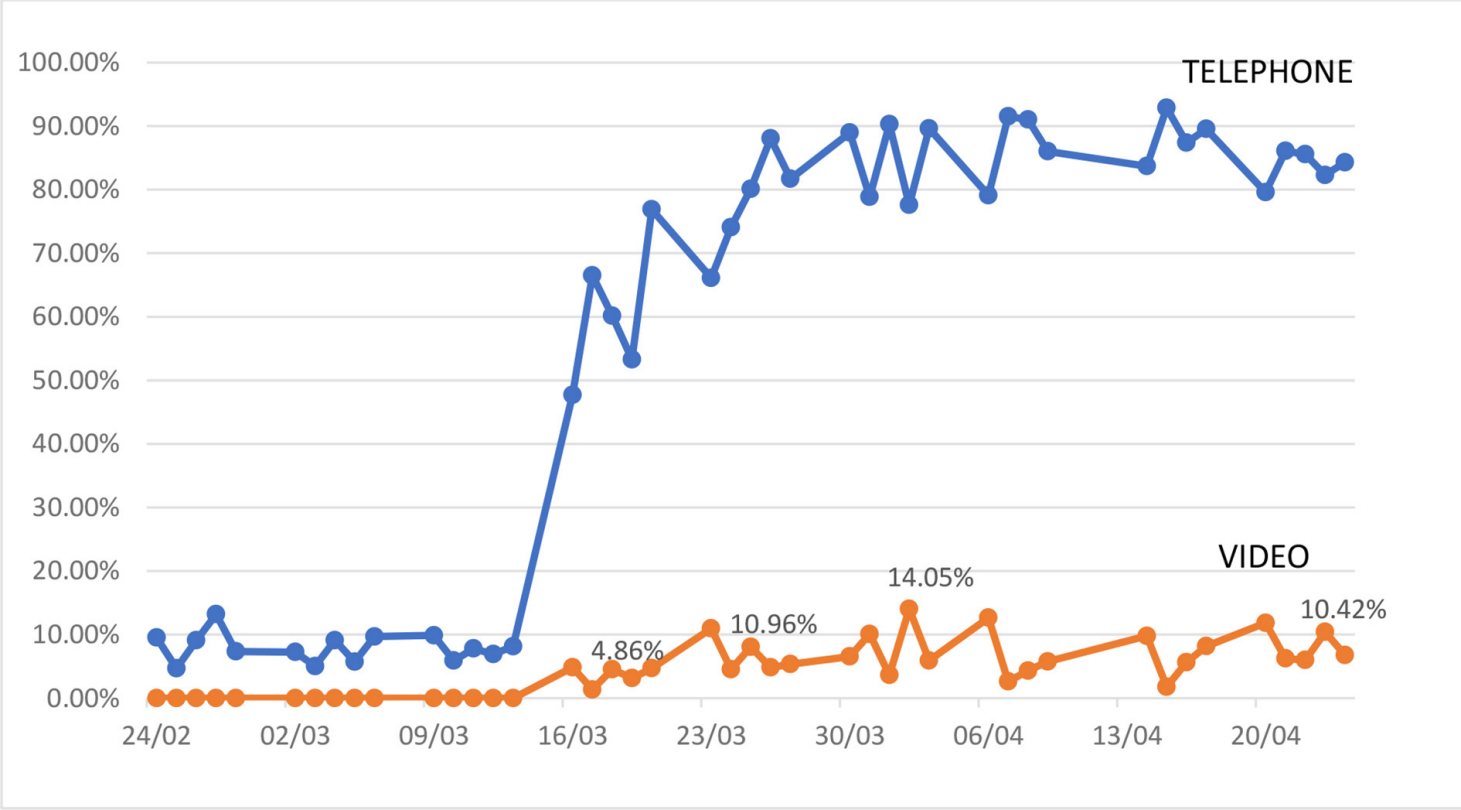
TEL and VID clinics - proportion of total virtual outpatient activity.

Figure 1 and table 2 demonstrate the per cent change of the different consultation types to achieve the target 80% VC, with the majority of VCs conducted using TEL. figure 2 demonstrates the proportion of virtual outpatient activity.

**Table 2:** Summary of results

	Baseline week 1 (w/c 2 March 2020)	Baseline week 2 (w/c 9 March 2020)	VC week 1 (w/c 16 March 2020)	VC week 2 (w/c 23 March 2020)	VC week 3 (w/c 30 March 2020)	VC week 4 (w/c 6 April 2020)	VC week 5 (w/c 13 April 2020)	VC week 6 (w/c 20 April 2020)
% F2F (n)	92.73 (3634)	92.27 (3535)	(target 80% VC)	37.31 (529)	15.14 (194)	6.96 (82)	6.72 (88)	5.47 (62)	8.18 (133)
% VID (n)	–	–		3.80 (54)	6.71 (86)	8.40 (99)	6.26 (82)	6.00 (68)	8.18 (133)
% TEL (n)	7.27 (285)	7.73 (296)		58.89 (835)	78.14 (1001)	84.65 (998)	87.02 (1140)	88.53 (1003)	83.63 (1359)
Total consultations	3919	3831		1418	1281	1179	1310	1133	1625

F2F, face-to-face; TEL, telephone consultation; VC, virtual consultation; VID, video consultation; w/c, week commencing.

### End of clinic reviews and patient feedback

As outlined in table 1, a simple evaluation tool was developed to capture patient and clinician experience of virtual clinics from 16 March 2020. These results are demonstrated in table 3. Feedback was reviewed and discussed during daily COVID-19 Action Team meetings.

**Table 3:** Summary of feedback from end of clinic reviews

		Responses (n)	Mean satisfaction score	Range	Virtual clinic again?
Patient feedback	Phone	111	90/100	(30–100)	94% yes
	Video	104	85/100	(0–100)	44% yes
Clinician feedback	Phone	52	N/A	N/A	N/A
	Video	51	78/100	(0–100)	49% yes
Virtual clinic total		242	87/100	(0–100)	73%

N/A, not available; VC, virtual consultation.

Patient satisfaction scores were high (90/100) for both telephone and video consultations. However, patients were more likely to consider using phone consultations again after a phone appointment (94% of patients) than video consultation patients wanting a further video appointment (36% of patients).

Open-ended qualitative data provided an overview of some of the potential reasons for high satisfaction among patients. These included the following:

- The offer of an alternative to F2F during the COVID-19 pandemic.
- Reduced travel times.
- Reduced waiting times.
- Reduced impact of travel on symptoms.

**Figure 3:**
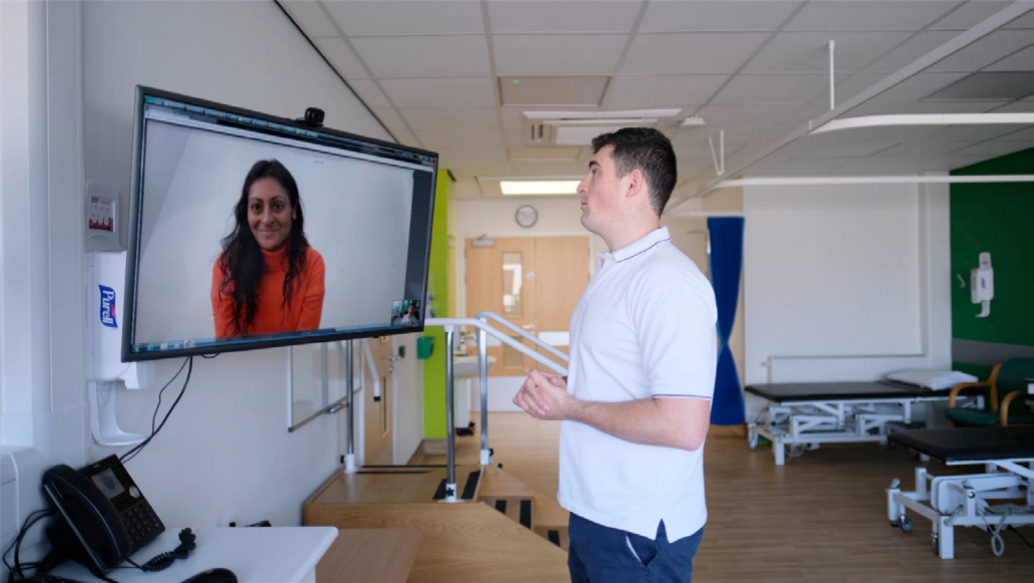
Video call between patient and clinician.

Open-ended qualitative data also provided an overview of some of the potential reasons for high satisfaction amongst patients and clinicians. These included the following:

- VC worked particularly well when the patient was already known to the clinician.
- VID was useful to assess a range of movements or visually assess a patient (figure 3).
- VC ran quicker than traditional F2F clinics.

Common reasons for poor satisfaction for both patients and clinicians included the following:

- Variable sound and picture quality.
- Low confidence levels with using the technology; both patients and clinicians required support with setting up the hardware and software.
- Equipment issues with outdated software (eg, using Internet Explorer or not having up-to-date phone/tablet/computer operating system software).
- Patients occasionally were left waiting in the virtual waiting area without having been acknowledged by the hospital.

These feedback forms were reviewed on a daily basis and informed the PDSA cycles.

Throughout the QI initiative, the COVID-19 Action Team collected information to support the future roll-out of VC after COVID-19. We now have in place all the technical elements to deliver outpatient appointments in a variety of ways appropriate to patient and clinician preference and convenience, and in the most effective way for the organisation. Prior to restarting a full outpatient service, a formal evaluation of patient and clinician experience will be undertaken. In addition, patient and staff stakeholder sessions will be held to inform any potential redesign of traditional outpatient models of care to incorporate VC.

## Discussion

The COVID-19 Action Team achieved the set goal of 80% VC by week 2. The use of QI methodology, specifically the use of repeated PDSA cycles, was essential to identify and overcome barriers to VC implementation.

The rapid implementation of VC was achieved due to the considerable resources directed to support it. The RNOH Senior Leadership Team clearly communicated VC as an important way to manage patients during the COVID-19 pandemic. The formation of the COVID-19 Action Team provided the initial resources to support patients and clinicians across the RNOH to engage with VC. The multidisciplinary nature of the COVID-19 Action Team, with the ability to draft in extra personnel as required, ensured a wide range of skills and abilities to respond to the dynamic and complex circumstances of implementation. While this QI initiative was delivered in one Trust across two sites, the lessons learnt are relevant in other healthcare settings (table 4).

**Table 4:** Lessons learnt regarding rapid implementation of virtual clinics

Lesson	Comment
It is important to have a multidisciplinary team when rapidly implementing VC.	The COVID-19 Action Team possessed a range of skills and abilities. The operational management and leadership provided the group with oversight of the workings of the RNOH and the strategic direction in response to COVID-19. Higher level support (from the Chief Operating Officer) facilitated engagement across RNOH. An assigned project manager directed the changes in response to the changing strategy of the Trust. QI personnel provided expertise on the change methodology required to facilitate a rapidly changing service. The use of QI provided a framework to identify and overcome unexpected issues. Insight from a clinical researcher helped identify potentially unexpected clinical issues. Access to data management support was essential to the success of the rapid implementation by providing real-time evaluation data. Flexibility across the group was essential to cross cover roles and responsibilities, particularly during the complex environment of COVID-19 when the system was undergoing rapid changes.
The presence of QI experts and the use of QI methodology facilitate rapid change.	The COVID-19 Action Team was strongly outcome-focused and action-focused, and the improvement expert was able to influence the approaches to ensure learning was captured and built on. A skilled improvement advisor added structure and form to the project while facilitating improvement at the pace required. The PDSA approach offered a pragmatic framework to build sustainable change.
It is important to have daily briefings across the team when rapidly implementing VC.	Daily virtual briefings with all members of the COVID-19 Action Team ensured optimal communication. Assigning a meeting chair and logging issues and actions ensured focus. Having all members of the multidisciplinary team present allowed for real-time troubleshooting and action planning.
It is important to have effective leadership when rapidly implementing VC.	The strategy of the RNOH was clearly communicated to members of the Trust community. Setting a timed and distinct goal provided staff with clear direction. The allocation of resources to facilitate the goal provided the community with the support to enact the goal.
The success of VC is reliant on engaged staff.	RNOH staff were flexible, proactive and supportive of the requirement to rapidly implement VC due to COVID-19. This supported a sense of common purpose, which was built on by project leads through continuously listening and reacting to issues raised by colleagues, leading to greater engagement and commitment to the shared goal.
The success of VC is reliant on adequate IT support.	The IT team rapidly rolled out a programme of software upgrades and installed hardware for VC across the RNOH within a short space of time. The IT team prioritised COVID-19-related tasks during this period.
The success of VC is reliant on adequate IG support.	The IG team were responsive to COVID-19 and provided clear and distinct guidance and troubleshooting for staff who were expected to work differently during this time.
The success of VC is reliant on adequate administrative support.	The admin teams responded quickly and effectively to the rapid implementation of VC due to COVID-19. The admin staff were required to call patients to inform them of changes to their care. The teams conducted a huge number of challenging conversations over a short space of time.
It is important to undergo regular evaluation when rapidly implementing VC.	After each consultation and at the end of each clinic, the feedback was studied, issues logged and communicated across the COVID-19 Action Team, and actions either taken immediately (eg, technical considerations) or agreed at the daily review meetings. These were conceived and presented as PDSA cycles.
Creating narrative through effective communications.	Effective staff and patient communications were central to the success of the project. Staff were supported to share their stories early on, alongside creating easily accessible technical advice and training materials. Examples include clinician blogs, a patient video, training webinars, highlights via existing executive updates, podcast, and use of intranet and internet to access up-to-date tools.

IG, information governance; IT, information technology; PDSA, Plan-Do-Study-Act; QI, quality improvement; RNOH, Royal National Orthopaedic Hospital; VC, virtual consultation.

The NHS Long Term Plan clearly sets out digital requirements needed to support NHS services.^7^ VC features heavily on the RNOH Trust objectives, with VC due to be phased in. The catalyst for VC implementation was the COVID-19 pandemic. This unique situation required urgency to rapidly implement these changes; patients and staff were largely understanding of the necessity for VC and grateful for the swift response to the pandemic.

The RNOH is dedicated to supporting the use of VC, in accordance with the NHS Long Term Plan.^7^ The key focus of the COVID-19 Action Team was to implement VC at pace. A secondary objective was to collect data to support the design of a substantive legacy of VC. Further stakeholder engagement initiatives and the use of frameworks^12^ or theories of implementation^13^ will support this. As we have found in this QI initiative, multidisciplinary working is key.

The majority of patients who underwent VC elected for a phone call (TEL) rather than a video call (VID). TELs have previously been found to be equally clinically effective as usual care,^14^ although TELs were associated with lower patient satisfaction. A qualitative interview study with participants from the PhysioDirect telephone and advice service^15^ found that the telephone service was broadly acceptable, but it was described as ‘impersonal’ and many were sceptical about the ability of TELs to achieve the goal of the session. For many the PhysioDirect service provided a ‘route in’ to care. The satisfaction of phone calls in our project was high (90/100), and approximately 94% indicated they would prefer a F2F call in the future. While TEL has proven to be a useful way to manage patients during the COVID-19 pandemic, further work needs to be done to understand its effectiveness and acceptability at the RNOH in the future.

Previous research^9^ at the RNOH investigating patient preferences for types of appointments found that approximately 50% of patients found the use of Skype to be acceptable for a follow-up consultation. These preferences were situational and fluid; patients stated they might choose VID or F2F under differing circumstances. The COVID-19 pandemic is a situation which has forced patients to undergo VC regardless of their preferences. Of those who underwent VC in our project, approximately half indicated they would prefer F2F for their next appointment. This is in keeping with a report^16^ that found that, from a survey of 2000 people, 55% would be willing to have a consultation for advice on an ongoing problem. Further research at the RNOH into preferences will likely sustain a legacy of clinically appropriate and acceptable VCs.

Greenhalgh *et al* 17 found that videoconferencing consultations appeared to work better when the patient and clinician knew each other. It is not obvious from our early evaluation data whether or not this is the case in our project, however, informal feedback from some clinicians indicates that having a prior relationship with the patient may have enhanced the consultation. Technical challenges have previously been shown to be prohibitive,^17^ and those encountered in our project occasionally led to abandonment of VID. Clinicians often responded to these issues by abandoning the VID and transferring to TEL, or in cases where it was the sound that was mainly disrupted, they spoke over TEL while capturing images from VID to enable an assessment. Individual agency and reflexive monitoring played an important part in the successful implementation of VC.^18^

Significant resources were intensively deployed to deliver this rapid implementation of VC. They included three additional members of staff almost full time to support the roll-out, the cost of hardware, software, information technology and telephone infrastructure. These costs are rarely reported in the literature^17^ and will need to be taken into account when commissioning digitally supported services in the future. Virtual clinics offer potential savings to the NHS which need to be further scoped. Savings for patients included the reduction in time spent travelling and the cost of travelling.

VC is not a novel approach to delivering outpatient appointments in healthcare, but this paper discusses an extremely rapid adoption. To our knowledge, this is the first report of an NHS hospital evaluating rapid implementation of VCs due to COVID-19.

The findings must be interpreted in light of their limitations. This was not a research project but a rapid evaluation of VC implementation. The pace of change led to some missing data which were manually collected wherever possible. The pragmatic approach described here does not seek to test or demonstrate statistical significance. Future research studies evaluating the effectiveness and acceptability of VC are required, particularly as services return to a ‘new normal’ after COVID-19.

Commitment from clinicians and administrators was initially due to the unusual circumstances of COVID-19 and the imperative to stop all non-essential F2F work, but engagement was maintained by continuous multichannel communications throughout the project. The future goal is to maintain a clinically appropriate level of VC post-COVID-19; the improvement-driven approach described in this paper has led to wide engagement, a clear plan of action and objective data to support this aim.

The implementation was within an orthopaedics setting; however, the findings from this report have been reported in a way to be as general as possible to allow for transportability.

## Conclusion

This QI initiative demonstrates that rapid implementation of virtual clinics can be achieved in response to the COVID-19 pandemic. The rapid implementation of VCs required a dedicated multidisciplinary team, expertise in operational management, QI, clinical care and data analysis. It required whole systems support from the RNOH Senior Leadership Team, information technology team, information governance team, administrative teams and clinical staff. This is a pragmatic QI initiative that was conducted at pace and must be considered in light of its limitations. To our knowledge this is the first report of rapid implementation of VCs across an NHS Hospital Trust conducted as a consequence of COVID-19. The findings from this report will be of interest to healthcare organisations looking to convert F2F clinics to virtual clinics. A structured and planned approach using QI methodology will be required to facilitate a return to F2F clinics as the COVID-19 situation allows.

## References

[1] WHO World health organisation Corobovirus disease 2019 (COVID-19) situation Report-51 2020. Available: https://www.who.int/docs/default-source/coronaviruse/situation-reports/20200311-sitrep-51-covid-19.pdf?sfvrsn=1ba62e57_10 [Accessed 29 Mar 2020].

[2] He F, Deng Y, Li W Coronavirus disease 2019 (COVID‐19): what we know? J Med Virol 2020 10.1002/jmv.25766 PMC7228340 32170865

[3] England PH Number of coronovirus (COVID-19) cases and risk in the UK 2020. Available: https://www.gov.uk/guidance/coronavirus-covid-19-information-for-the-public [Accessed 29 Mar 2020].

[4] Greenhalgh T, Koh GCH, Car J Covid-19: a remote assessment in primary care. BMJ 2020;368:m1182. 10.1136/bmj.m1182 32213507

[5] Greenhalgh T, Wherton J, Shaw S, et al Video consultations for covid-19. BMJ 2020;368:m998 10.1136/bmj.m998 32165352

[6] NHSX Covid-19 information governance advice for health and care professionals 2020, 2020 Available: https://www.nhsx.nhs.uk/key-information-and-tools/information-governance-guidance/health-care-professionals [Accessed 29 Mar 2020].

[7] NHS The NHS long term plan. Health Do, 2019.

[8] Gilbert AW, Jaggi A, May CR What is the patient acceptability of real time 1:1 videoconferencing in an orthopaedics setting? A systematic review. Physiotherapy 2018;104:178–86. 10.1016/j.physio.2017.11.217 29361298

[9] Gilbert AW, Jaggi A, May CR What is the acceptability of real time 1:1 videoconferencing between clinicians and patients for a follow-up consultation for multi-directional shoulder instability? Shoulder Elbow 2019;11:53–9. 10.1177/1758573218796815 30719098 PMC6348581

[10] Gilbert AW, Jones J, Stokes M, et al Protocol for the connect project: a mixed methods study investigating patient preferences for communication technology use in orthopaedic rehabilitation consultations. BMJ Open 2019;9:e035210. 10.1136/bmjopen-2019-035210 PMC6924859 31831552

[11] Langley GL, Moen R, Nolan KM, et al The improvement guide: a practical approach to enhancing organizational performance. 2nd edn San Francisco: Jossey-Bass Publishers, 2009.

[12] Greenhalgh T, Wherton J, Papoutsi C, et al Beyond adoption: a new framework for theorizing and evaluating nonadoption, abandonment, and challenges to the scale-up, spread, and sustainability of health and care technologies. J Med Internet Res 2017;19:e367. 10.2196/jmir.8775 29092808 PMC5688245

[13] May CR, Johnson M, Finch T Implementation, context and complexity. Implement Sci 2016;11:141. 10.1186/s13012-016-0506-3 27756414 PMC5069794

[14] Salisbury C, Montgomery AA, Hollinghurst S, et al Effectiveness of PhysioDirect telephone assessment and advice services for patients with musculoskeletal problems: pragmatic randomised controlled trial. BMJ 2013;346:f43. 10.1136/bmj.f43 23360891 PMC3558547

[15] Pearson J, Richardson J, Calnan M, et al The acceptability to patients of PhysioDirect telephone assessment and advice services; a qualitative interview study. BMC Health Serv Res 2016;16:104–04. 10.1186/s12913-016-1349-y 27020840 PMC4810506

[16] Castle-Clarke S What will new technology mean for the NHS and its patients? four big technological trends, 2018 Available: https://www.nuffieldtrust.org.uk/files/2018-06/1530028974_the-nhs-at-70-what-will-new-technology-mean-for-the-nhs-and-its-patients.pdf

[17] Greenhalgh T, Shaw S, Wherton J, et al Real-World implementation of video outpatient consultations at macro, meso, and micro levels: Mixed-method study. J Med Internet Res 2018;20:e150. 10.2196/jmir.9897 29625956 PMC5930173

[18] May C, Finch T, Implementing FT Implementing, embedding, and integrating practices: an outline of normalization process theory. Sociology 2009;43:535–54. 10.1177/0038038509103208

[19] Letocha J RNOH virtual outpatient clinics: a patient guide (Royal national orthopaedic Hospital version), 2020 Available: https://youtu.be/1aL-WFe82xo [Accessed 31 Mar 2020].

[20] Letocha J Nhs virtual outpatient clinics: a patient guide (NHS version), 2020 Available: https://youtu.be/M39BPLy9vFE [Accessed 31 March 2020].

